# Consistency between 3 days' dietary records and 24-h urine in estimating salt intake in children and adolescents

**DOI:** 10.3389/fpubh.2022.1071473

**Published:** 2022-12-23

**Authors:** Jie Dong, Xiaoran Yu, Xun Li, Shiting Xiang, Yongquan Qin, Shaolun Zhu, Jie Zheng, Yinkun Yan

**Affiliations:** ^1^Pediatrics Research Institute of Hunan Province, Hunan Children's Hospital, Changsha, China; ^2^Department of Center for Non-communicable Disease Management, Beijing Children's Hospital, National Center for Children's Health, Capital Medical University, Beijing, China; ^3^The Middle School of Pantang, Taoyuan, China; ^4^The Middle School of Fengshu, Taoyuan, China; ^5^The Primary School of Qinglin, Taoyuan, China

**Keywords:** sodium, 24-h urine, consistency, children and adolescents, weighed dietary record

## Abstract

**Purpose:**

This study aimed to evaluate the salt intake in boarding school students and the consistency between salt intake measurements based on 24-h urine and weighed dietary records over 3 consecutive days in this population.

**Methods:**

This was a school-based cross-sectional study. Overweight (including obesity) or hypertensive students aged 6–14 years and their normal counterparts were recruited for this study at three boarding schools in China. Three consecutive 24-h urine samples were collected from all participants. During the collection period of 24-h urine, the weighed diet records were collected in children who had all three meals at the school canteens on weekdays. Incomplete 24-h urine or dietary records were excluded from the analysis.

**Results:**

The median salt excretion was 6,218 [4,636, 8,290] mg by 24-h urine and 120 (82.2%) consumed excess salt among the participants. The median salt intake was 8,132 [6,348, 9,370] mg by dietary records and 112 (97.4%) participants consumed excess salt than recommended in participants who have all three meals in the school canteens. In children with complete dietary records and 24-h urine, the level of salt intake estimated by 24-h urine accounted for 79.6% of the dietary records.

**Conclusion:**

Our study showed that boarding school students consumed excessive salt from school canteens. Thus, policies or strategies targeting school canteens are urgently needed. Weighed dietary records are recommended if feasible.

## 1. Introduction

A high-salt diet is a major risk factor contributing to 44 million disability-adjusted life years (DALYs) and 1.8 million deaths worldwide in 2019 and was the third-most important risk factor of DALYs and deaths among Chinese adults in 2017 (1, 2). Health organizations recommend consuming <5 g of salt per day in adults (3, 4), although consumption by individuals in many countries far exceeds this level (5–7). High salt intake is also common among children and adolescents, similar to adults (8–10). To reduce the heavy health burden caused by high dietary salt intake, several large-scale intervention studies have been undertaken in China (11–13), with two studies conducted in urban children (14, 15). An important premise for these interventional studies is the accurate estimation of salt intake at the individual level.

Currently, the measurement of salt intake from 24-h urine samples is considered the gold standard for individual-level evaluations. Although studies conducted in strictly controlled environments have revealed that at least 3 days' 24-h urine records are needed and that a single 24-h urine result is no better than a coin flip in estimating salt intake at individual levels (16, 17), many studies were based on a single 24-h urine sample due to the heavy participant burden (11, 13). For boarding school students who have all three meals in school canteens on weekdays, weighed dietary records are a feasible and reliable approach to assess the level of salt intake. Although boarding schools are common in rural areas and there are over 26 million boarding school students (aged 7–15 years) in China (18), few studies have reported the level of salt intake in this young population using weighed dietary records or compared the differences between salt intake measurements obtained with 24-h urine and weighed dietary records in this convenient population. Therefore, this study aimed to evaluate the salt intake in boarding school students and the consistency between salt intake measurements based on 24-h urine and weighed dietary records over 3 consecutive days in this population.

## 2. Materials and methods

### 2.1. Participants

This cross-sectional study was conducted at three boarding schools, two primary schools (grades 1–6), and one junior high school (grades 7–8), located in the rural areas of Hunan Province, China, from September to October 2021. This survey aimed to explore the interactions of salt, overweight (including obesity) or hypertension, and gut microbiota and to evaluate the consistency between salt intake assessments based on dietary and 24-h urine records in children. Overweight or obese students from the three schools were recruited for this study. Students from grades 5 to 6 who were hypertensive after three separate blood pressure measurements were also recruited. For each overweight or hypertensive participant, a sex- and grade-matched control student with normal weight or blood pressure was included in this study. We excluded students with current diarrhea or fever and girls in menstruation from this study. After obtaining written informed consent from the participants and their guardians, a total of 180 students were recruited for the study. The study was conducted in accordance with the guidelines of the Declaration of Helsinki and approved by the Hunan Children's Hospital Review Board (KYSQ2021-212, HCHLL-2022-77).

### 2.2. 24-h urine collection

Three consecutive 24-h urine samples were collected from all participants. Before collection, detailed face-to-face instructions were provided to all participants. The start time of the 24-h urine collection was recorded after the participants emptied their bladders, and the participants were then provided with a 2-L wide-mouth container and a urine collection aid. The finish time was recorded at the same time on the second or third day after the participants had passed their last urine in the container. Another urine container was provided to the participants upon completion of the first or second 24-h urine collection. The 24-h urine samples were evenly mixed, and 2 ml aliquots were extracted within 2 h, temporarily stored at −20°C, and then transported to a centralized laboratory within 2 months.

Concentrations of urinary sodium, potassium, and creatinine were measured at the Laboratory Center of Hunan Children's Hospital using the ion-selective electrode method (sodium and potassium) and enzymatic method (creatinine) (Beckman AU-5800).

### 2.3. Weighed dietary records

3-day dietary records of the participants from grades 5 to 8 were obtained during the period of 24-h urine collection. The records included the weighed intake from the cafeteria and the participant-reported intake of snacks. During the preparation of each meal in the school canteens, the amounts of salt and other salty seasonings used were weighed and recorded. The sodium concentration in each food item was calculated as the amount of sodium divided by the net weight of the food item after cooking. For each participant, the researchers weighed each food item before the meal and the leftovers of each food item after the meal. The sodium intake of each food item was calculated as the sodium concentration (mg/100 g) multiplied by the net intake (g). The participants were also asked to record the details of snacks taken beside the meals (such as pickles, salty snacks, beverages, and biscuits), including the time of intake, snack name or brand, net weight, and source of the snacks (homemade or store-bought). If the net weights of the recorded snacks were missing, the net weight of the same or similar snacks sold in the school shop or shops around the school was used as a substitute. The sodium concentration of the snacks was determined from the values for the same or similar snacks sold in the school shop or shops around the school.

### 2.4. Statistical analysis

Incomplete 24-h urine was defined as follows: (1) urinary volume <300 mL; (2) >1 instance of leakage of urine; or (3) 24-h urine creatinine level <2.0 mmol for boys and <1.2 mmol for girls. Children with 2 or 3 days' complete 24-h urine were included in the urinary analysis. Incomplete dietary records were defined as follows: (1) missing one or more meals at the school cafeteria and (2) not handing in the snack-record sheet at the end of the collection period. Children with complete dietary records for 2 or 3 days were included in the dietary analysis. An average of 2 or 3 days' complete 24-h urine or dietary records was used for the analyses. The cutoffs for high salt and inadequate potassium intake were adjusted downward for children aged 6–14 years based on their energy requirements relative to adults. Absolute differences between salt intake measured using the averaged 24-h urine and dietary records, between salt intake measured using the complete 24-h urine record on day 2 and the complete dietary record on day 1, and between salt intake measured using the complete 24-h urine record on day 3 and the complete dietary record on day 2 were calculated and classified into three groups: ≤1, 1–3, and >3 g.

Weight and height were measured, and body mass index (BMI) was categorized as normal, overweight, or obese according to the International Obesity Task Force (IOTF) standards (19). Blood pressure (BP) was measured using validated devices (Omron HBP-1300) (20) and a standard procedure (21), and BP status was classified according to the reference values for Chinese children (22). Continuous variables were expressed as mean ± standard deviation or median and interquartile range. A *t*-test or rank-sum test was used to test the differences between the two groups. Categorical variables were presented as numbers and proportions. All analyses were conducted using SPSS 20.0, and a two-sided *P* < 0.05 was considered statistically significant.

## 3. Results

The participants' basic characteristics are listed in Table 1. Among the 180 participants (mean age, 10.9 ± 1.5 years; range: 6–14 years) who agreed to participate in this study, 128 (71.1%) were boys, 48 (26.7%) were overweight and 52 (28.9%) were obese, and 18 (10%) were hypertensive after BP measurement on three separate occasions.

**Table 1:** Basic characteristics of all participants (*n* = 180).

**Indicators**	**All**	**School 1 (G5–6)**	**School 2**		**School 3 (G7–8)**
			**G1–4**	**G5–6**	
*N*	180	54	34	60	32
Sex, boy	128 (71.1)	36 (66.7)	28 (82.4)	32 (53.3)	32 (100)
Age, year	10.9 ± 1.5	11.1 ± 0.6	8.6 ± 1.1	11.1 ± 0.6	12.9 ± 0.6
Weight, kg	46.3 ± 14.2	48 ± 13.3	34.2 ± 10.3	47 ± 11.0	55.1 ± 16.6
Height, cm	146.8 ± 10.8	148.2 ± 8	132.8 ± 7.1	148.1 ± 7.4	156.7 ± 9.2
BMI, kg/m^2^	21.1 ± 4.7	21.6 ± 4.8	19.1 ± 4.4	21.2 ± 4.2	22.1 ± 5.2
SBP, mmHg	109.7 ± 12.0	114.8 ± 11.1	101.9 ± 7.9	108.7 ± 11.0	111.2 ± 14.4
DBP, mmHg	64.6 ± 8.0	67.8 ± 9.4	61.1 ± 6.2	64.7 ± 7.3	62.9 ± 6.6
HR, beats/min	75.8 ± 16.9	73.2 ± 17.3	71.8 ± 16.4	78.2 ± 19.5	80.1 ± 8.0
**BMI status**					
Normal	80 (44.4)	22 (40.7)	17 (50.0)	24 (40.0)	17 (53.1)
Overweight	48 (26.7)	14 (25.9)	5 (14.7)	22 (36.7)	7 (21.9)
Obesity	52 (28.9)	18 (33.3)	12 (35.3)	14 (23.3)	8 (25.0)

School 1, FX; School 2, PX; School 3, PZ. SBP, systolic blood pressure; DBP, diastolic blood pressure; HR, heart rate; BMI, body mass index.

Table 2 shows the results of the 3-day 24-h urine tests. A total of 173, 172, and 169 participants provided 24-h urine samples on the first, second, and third days, respectively, of which 137, 139, and 139 participants provided complete 24-h urine samples, respectively. Overall, 3- or 2-day complete 24-h urine records were available for 146 participants, and their average salt excretion was 6,218 [4,636, 8,290] mg, with the salt levels exceeding the recommended level in 120 (82.2%) students (cutoff values are provided in Supplementary Table 1).

**Table 2:** Salt excretion from 24-h urine.

	**All**	**School 1 (G5–6)**	**School 2**		**School 3 (G7–8)**
			**G1–4**	**G5–6**	
**The 1st 24 h urine**					
N	137	41	25	43	28
Volume, ml	550 [428, 789]	595 [455, 848]	555 [420, 763]	540 [380, 715]	533 [456, 809]
Cna, mmol/L	161.1 [122.3, 202.3]	173 [126, 208]	137.8 [89.5, 190.4]	151 [95.3, 176.9]	182 [152, 214]
Ck, mmol/L	25.5 [19.9, 32.2]	29 [22, 38]	23.5 [18, 31.8]	25.9 [21.6, 33]	22 [16,17]
Ccr, umol/L	1,0450 [7,273, 14,262]	9,713 [6,435, 12,578]	7,326 [6,298, 9,334]	12,803 [9,343, 15,631]	13,795 [10,178, 18,329]
Sodium, mg	2,002 [1,408, 2,910]	2,422 [1,711, 3,460]	1,715 [1,333, 2,157]	1,572 [1,248, 2,775]	2,240 [1,725, 3,213]
Salt, mg	5,092 [3,581, 7,403]	6,160 [4,351, 8,799]	4,362 [3,390, 5,487]	3,998 [3,174, 7,057]	5,698 [4,388, 8,171]
K, mg	571 [428, 739]	737 [562, 919]	544 [459, 699]	549 [435, 712]	447 [353, 567]
Cr, mmol	5.91 [4.34, 7.96]	5.83 [4.77, 7.15]	3.90 [3.26, 4.63]	6.22 [5.13, 8.71]	8.24 [6.13, 9.16]
**The 2nd 24 h urine**					
*N*	139	49	21	40	29
Volume, ml	625 [465, 835]	670 [485, 893]	600 [515, 730]	534 [408, 802]	740 [497, 940]
Cna, mmol/L	167.3 [127.7, 220.7]	193 [147, 227]	151.6 [127.2, 183.2]	130.9 [90.6, 216.5]	194 [157, 230]
Ck, mmol/L	25.7 [17.8, 31.3]	27 [21,30]	30.7 [21.3, 38.1]	26.3 [16.6, 32.7]	21 [15,30]
Ccr, umol/L	9,311 [7,112, 13,374]	8,565 [6,851, 10,523]	7,550 [6,777, 11,057]	11,138 [7,952, 16,102]	12,502 [8,542, 18,661]
Sodium, mg	2,402 [1,847, 3,324]	2,788 [2,261, 3,738]	2,011 [1,669, 2,514]	1,837 [1,184, 2,281]	3,324 [2,411, 4,043]
Salt, mg	6,110 [4,698, 8,455]	7,091 [5,750, 9,509]	5,115 [4,244, 6,395]	4,671 [3,012, 5,802]	8,455 [6,132, 10,284]
K, mg	581 [465, 728]	705 [501, 842]	632 [584, 759]	548 [452, 633]	561 [446, 722]
Cr, mmol	6.10 [4.60, 7.95]	5.64 [4.09, 6.59]	4.72 [4.10, 5.51]	6.83 [4.75, 7.59]	9.05 [7.29, 11.18]
**The 3rd 24 h urine**					
*N*	139	49	21	40	29
Volume, ml	650 [475, 880]	650 [505, 838]	580 [425, 685]	530 [410, 765]	1,029 [685, 1,413]
Cna, mmol/L	179.5 [135.4, 241.2]	201 [144, 240]	166.4 [117.7, 209.2]	172.8 [110.7, 250.3]	178 [148, 242]
Ck, mmol/L	22.9 [17.2, 30.9]	24 [20, 34]	31.8 [23.1, 41]	24 [16.9, 29.9]	16 [12,21]
Ccr, umol/L	9,020 [6,738, 12,202]	8,501 [6,258, 11,712]	8,181 [6,215, 10,824]	10,319 [7,421, 16,214]	9,615 [7,308, 12,711]
Sodium, mg	2,488 [1,893, 3,693]	2,648 [2,045, 3,825]	1,961 [1,474, 2,493]	2,264 [1,693, 2,648]	4,495 [3,262, 5,762]
Salt, mg	6,327 [4,815, 9,394]	6,734 [5,201, 9,730]	4,988 [3,748, 6,342]	5,757 [4,306, 6,736]	11,432 [8,296, 14,654]
K, mg	596 [481, 743]	665 [531, 847]	621 [533, 893]	532 [411, 654]	535 [473, 721]
Cr, mmol	6.09 [4.65, 8.24]	5.48 [4.61, 6.83]	4.79 [3.79, 5.20]	6.55 [5.00, 7.67]	8.89 [7.58, 11.59]
**Average**					
*N*	146	51	23	41	31
Sodium, mg	2,445 [1,823, 3,259]	2,894 [2,147, 3,527]	1,858 [1,697, 2,541]	1,961 [1,474, 2,507]	3,335 [2,190, 4,218]
Salt, mg	6,218 [4,636, 8,290]	7,362 [5,461, 8,971]	4,726 [4,317, 6,464]	4,988 [3,749, 6,376]	8,483 [5,570, 10,729]
K, mg	589 [489, 714]	678 [579, 788]	616 [548, 736]	524 [449, 653]	517 [450, 698]
Excess salt	120 (82.2)	44 (86.3)	20 (87.0)	27 (65.9)	29 (93.5)
Inadequate K	146 (100)	51 (100)	23 (100)	41 (100)	31 (100)

School 1, FX; School 2, PX; School 3, PZ. Cna, concentration of sodium; Ck, concentration of potassium; Ccr, concentration of creatinine; K, potassium; Cr, creatinine.

3-day dietary records were collected from 146 participants in grades 5–8. Four participants did not hand in the snack-record sheets at the end of the collection period and were excluded from the dietary analysis. On the 1st day of dietary recording, 35 students missed one or more meals in the school cafeteria; thus, 107 1st-day records were analyzed. Similarly, 117 and 105 dietary records for the second and third days, respectively, were analyzed. The salt intake from the school cafeteria was 7,047 [5,440, 9,129] mg, 7,150 [5,520, 9,133] mg, and 7,789 [6,037, 9,907] mg on the first, second, and third days, respectively. Salt intake from snacks was 368 [0, 1,092] mg, 137 [0, 749] mg, and 0 [0, 406] mg on the first, second, and third days, respectively. For the 115 participants with complete dietary records, the total salt intake was 8,132 [6,348, 9,370] mg, and 95% [IQR: 91%-100%] of the salt was consumed in school canteens (Table 3). Three-day records of the salt and salty seasonings used in each food item in the three schools' cafeterias are provided in Supplementary Table 2, and the net intake and sodium intake for each food item in the three schools are provided in Supplementary Table 3.

**Table 3:** Salt intake from weighed dietary records^a^.

	**All**	**School 1 (G5–6)**	**School 2 (G5–6)**	**School 3 (G7–8)**
**Day 1**				
*N*	107	38	47	22
Salt from cafeteria, mg	7,047 [5,440, 9,129]	6,014 [5,088, 7,669]	8,537 [6,066, 9,874]	6,888 [5,691, 9,038]
Salt from snacks, mg	368 [0, 1,092]	879 [96, 1,945]	0 [0, 509]	388 [0, 993]
Total salt intake, mg	8,124 [6,200, 9,874]	7,385 [6,143, 9,406]	9,030 [6756, 10,221]	7,907 [6,025, 9,349]
Ratio^b^	0.95 [0.87, 1.00]	0.88 [0.74, 0.99]	1.00 [0.94, 1.00]	0.95 [0.87, 1.00]
**Day 2**				
*n*	117	40	49	28
Salt from cafeteria, mg	7,150 [5,520, 9,133]	9,217 [6,456, 10,560]	6,034 [4,242, 7,037]	8,187 [6,812, 9,445]
Salt from snacks, mg	137 [0, 749]	660 [0, 1,064]	0 [0, 388]	0 [0, 792]
Total salt intake, mg	7391 [6,001, 9,772]	10,114 [6,936, 11,467]	6,202 [4,478, 7,275]	8,615 [7,483, 9,735]
Ratio^b^	0.98 [0.90, 1.00]	0.93 [0.86, 1.00]	1.00 [0.94, 1.00]	1.00 [0.91, 1.00]
**Day 3**				
*n*	105	32	47	26
Salt from cafeteria, mg	7,789 [6,037, 9,907]	11,349 [8,241, 14,402]	6,277 [4,716, 7,642]	8,799 [7,638, 9,947]
Salt from snacks, mg	0 [0, 406]	0 [0, 708]	0 [0, 407]	0 [0, 378]
Total salt intake, mg	8,049 [6,427, 10,437]	11,585 [9,301, 14,481]	6,482 [4,716, 7,689]	9,044 [7,853, 10,385]
Ratio ^b^	1.00 [0.95, 1.00]	1.00 [0.95, 1.00]	1.00 [0.94, 1.00]	1.00 [0.96, 1.00]
**Average**				
*n*	115	39	49	27
Total salt intake, mg	8,132 [6,348, 9,370]	9,357 [7,707, 11,629]	6,828 [5,724, 8,390]	8,590 [7,678, 9,271]
Excess salt intake	112 (97.4)	39 (100)	46 (93.9)	27 (100)
Ratio^b^	0.95 [0.91, 1.00]	0.93 [0.83, 0.96]	0.98 [0.92, 1.00]	0.96 [0.92, 1.00]

a Data are presented as median [IQR, interquartile range] or *n* (%);

b ratio = salt intake from cafeteria/total salt intake. School 1, FX; School 2, PX; School 3, PZ.

For the 100 participants with complete 24-h urine collection and dietary records, the average salt intake was 8,392 [6,733, 9,471] mg from dietary records and 6,362 [4,731, 9,153] mg from 24-h urine records, with a median ratio (salt assessed by 24-h urine records divided by salt assessed by dietary records) of 0.796 [IQR: 0.652–1.046]. In the three schools, the median differences in salt intake between the dietary and 24-h urine records were 1,955 [714, 3,423] mg, 1,521 [566, 2,776] mg, and −1,485 [−1,946, 1,923] mg, respectively (Table 4). The proportions of absolute differences in the average salt estimation between the 24-h urine and dietary records within 1 g, 1–3 g, and >3 g were 17, 61, and 22%, respectively (Table 5).

**Table 4:** Consistency between averaged dietary record and 24-h urine in estimating salt intake, mg^a^.

	***n***	**Dietary record**	**24-h urine**	**Difference**		**Ratio^b^**
				**Median [IQR]**	**Mean [95%CI]**	
Averaged	100	8,392 [6,733, 9,471]	6,362 [4,731, 9,153]	1,435 [−441, 2,648]^*^	1,388 [906, 1,869]^*^	0.80 [0.65, 1.05]
Day 1	85	8,158 [6,628, 9,979]	5,377 [3,689, 7,537]	2,163 [194, 4,938]^*^	2,407 [1,634, 3,179]^*^	0.70 [0.46, 0.96]
Day 2	97	8,094 [6,300, 10,114]	6,444 [4,837, 9,044]	1,471 [−325, 3,102]^*^	1,372 [860, 1,884]^*^	0.82 [0.61, 1.04]
Day 3	88	8,479 [6,476, 11,048]	6,764 [5,324, 10,652]	879 [−2,009, 2,966]	718 [−167, 1,603]	0.87 [0.68, 1.24]
School 1	38	9,408 [7,665, 11,753]	7,064 [5,641, 9,305]	1,955 [714, 3,423]^*^	2,199 [1,347, 3,052]^*^	0.77 [0.64, 0.92]
School 2	36	6,799 [5,721, 8,437]	4,922 [3,901, 6,348]	1,521 [566, 2,776]^*^	1,671 [1059, 2282]^*^	0.75 [0.61, 0.90]
School 3	26	8,601 [7,871, 9,324]	9,494 [5,700, 11,185]	−1,485 [−1,946, 1,923]	−191 [−1,102, 721]	1.16 [0.73, 1.22]

a Data are presented as median [IQR] unless specified,

b Ratio = salt from 24-h urine/salt from dietary record,

* *P* < 0.05. School 1, FX; School 2, PX; School 3, PZ.

**Table 5:** Absolute difference distribution of salt estimation between dietary records and 24-h urine^a^.

	**≤1 g**	**(1, 3] g**	**>3 g**
Total	17 (17.0)	61 (61.0)	22 (22.0)
2nd 24-h urine-−1st dietary records^b^	16 (16.7)	43 (43.9)	37(38.5)
3rd 24-h urine-−2nd dietary records^c^	26 (26.5)	40 (40.8)	32 (32.7)

a Data are presented as *n* (%);

b *n* = 96;

c *n* = 98.

## 4. Discussion

Our study is the first to explore salt intake in children and adolescents by using 3-day 24-h urine and dietary records. The average salt excretion assessed by 24-h urine records was 6218 [4636, 8290] mg in 146 participants with ≥2 days of complete 24-h urine records. The average salt intake assessed by dietary records was 8,132 [6,348, 9,370] mg in 115 participants from Grades 5–8 with ≥2 days of complete dietary records. In the 100 participants with both complete 24-h urine and dietary records, the level of salt intake estimated by the 24-h urine records accounted for 79.6% of the level estimated by dietary records.

The median salt excretion based on the 24-h urine records was similar between this study and our previous study conducted in another school in 2017 (8) (Grade 5–6: 6,146 vs. 6,192 mg; Grade 7–8: 8,483 vs. 7,236 mg). The level of salt intake in our population was also similar to the baseline salt intake in a randomized trial conducted on Chinese urban children in 2018 (5.3 ± 1.8 g vs. 5.5 ± 2.0 g) (9). A systematic review and meta-analysis showed that the mean salt excretion in the 24-h urine record was 8,839 mg in Chinese children aged 6–16 years (23), which was higher than that in our results. However, most studies included in that meta-analysis were based on single 24-h urine samples (23), with only one study based on two consecutive 24-h urine samples (24), which may have resulted in a high risk of random errors. Children from other countries also consumed much more salt than recommended (10, 25, 26), which indicated that attention and effective interventions are urgently warranted in this young population.

Assessment of individual-level salt intake using an accurate and reliable method is vital, especially in interventional studies. Assessments based on 24-h urine records are widely acknowledged as the gold standard, and over 92% of the salt can be recovered in the urine. One study conducted on 84 children in New Zealand also showed that 24-h urine records can provide an adequate measurement of sodium intake (27). The similar levels of salt intake estimated by the 3 days' 24-h urine collection records in our population and by 1 or 2 days' 24-h urine collection in other studies suggest that salt intake at the population level can be accurately estimated by single or multiple 24-h urine collections. However, studies conducted in adults have shown that seven 24-h urine collections are needed to achieve 90% accuracy in estimates of salt intake (16, 17), and for individuals on a high-salt diet, at least 10 repeated 24-h urine collections are required to reach 75% reliability (28). In comparison with the average of three complete 24-h urine records, an absolute difference of ≤1 g in salt intake measured by 1 day's 24-h urine record was only present in ~50% of the participants in our study, suggesting a high risk of random errors and indicating that a single 24-h urine sample was not enough to estimate salt intake at individual levels.

Another problem identified in this study is the major source of salt and the development of intervention strategies to reduce salt intake. Processed food is a major source of salt intake in developed countries (10, 29, 30), and interventions targeted at food industries have been shown to reduce salt intake and BP (31). Approximately 67–75% of salt, however, is added by the consumer during home cooking in China (32, 33). Our results showed that up to 95% of the salt was consumed by boarding school students in the school canteen during weekdays. Two large-scale intervention studies targeted Chinese urban children and focused on health education (14, 24). To the best of our knowledge, there are no studies or policies that regulate salt usage or limit salt concentration in school canteens. Our results showed that salt concentrations varied greatly among school canteens (Supplementary Table 1), which directly contributed to the high salt intake by schoolchildren. Over 26 million young children live in boarding schools and have meals in canteens in rural China; thus, interventions or policies targeted at salt usage in school canteens are urgently required and would make a significant contribution to salt reduction in children.

The strengths of our study included the analysis based on 3 days' 24-h urine collection and weighed dietary records, with 3 days' complete 24-h urine records obtained for 103 (70.5%) participants, 2 days' complete 24-h urine records obtained for 43 (29.5%) participants, 3 days' complete dietary records available for 91 (79.1%) participants, and 2 days' complete dietary records available for 24 (20.9%) participants. Moreover, quality control was performed by the same researcher during the entire collection period, and 10% of duplicate urine samples were tested in parallel, with the biochemist who performed the measurements blinded to the identity of the duplicate samples.

Our study had some limitations, particularly in relation to the representativeness of the study population. About half of our participants were overweight or obese and about 10.0% were hypertensive based on three separate BP measurements, and the proportions of boys in our population for assessment of the estimated salt intake may have been higher than that in the general population. However, the differences in salt intake estimated by dietary records in different weight/BP/sex groups were not statistically significant for our population (data not presented). Another limitation was that food items consumed outside the school cafeteria (including snacks, processed vegetables, or homemade salted vegetables) were not weighed. We recorded the salt density and net weight of snacks of the same brand name sold in stores near the school. However, the salt content in sugars, fruits, and sugar-sweetened beverages was not calculated, and the lowest salt density and net weight were calculated if there were several snacks with similar names. Therefore, the salt intake from snacks may have been underestimated.

In conclusion, our study showed that boarding school students consumed excessive salt from school canteens. Thus, policies or strategies targeting school canteens are urgently needed. Salt excreted from 24-h urine accounted for 79.6% of the salt assessed by dietary records over 3 consecutive days in children, and a single 24-h urine sample was insufficient for individual-level estimations of salt intake. Thus, weighed dietary records are recommended if feasible.

## Data availability statement

The raw data supporting the conclusions of this article will be made available by the authors, without undue reservation.

## Ethics statement

The studies involving human participants were reviewed and approved by Hunan Children's Hospital Review Boards. Written informed consent to participate in this study was provided by the participants' legal guardian/next of kin.

## Author contributions

Conceptualization, writing, reviewing, and editing: JD and YY. Methodology: XY, SX, and XL. Formal analysis and writing the original draft preparation: JD. Investigation: JD, YQ, SZ, and JZ. Resources: YQ, SZ, and JZ. All authors have read and agreed to the published version of the manuscript.
