# Supplementary material for: Consistency between 3 days' dietary records and 24-h urine in estimating salt intake in children and adolescents
Source: Front Public Health. 2022 Dec 23;10:1071473. doi: 10.3389/fpubh.2022.1071473 (PMC9822649; doi:10.3389/fpubh.2022.1071473)
Supplement: Supplementary file 1 [file Table_1.DOCX]

Supplemental Table 1. Cutoffs of high salt and inadequate potassium for children aged 6-14 years.

| Age, year | Boy | | |  | Girl | | |  |
| --- | --- | --- | --- | --- | --- | --- | --- | --- |
|  | Energy requirement, kcal/day | Salt RNI, g | Potassium RNI, g |  | Energy requirement, kcal/day | Salt RNI, g | Potassium  RNI, g | |
| 6 | 1600 | 3.1 | 2.2 |  | 1450 | 3.5 | 2.4 | |
| 7 | 1700 | 3.3 | 2.3 |  | 1550 | 3.7 | 2.6 | |
| 8 | 1850 | 3.6 | 2.5 |  | 1700 | 4.0 | 2.8 | |
| 9 | 2000 | 3.8 | 2.7 |  | 1800 | 4.3 | 3.0 | |
| 10 | 2050 | 3.9 | 2.8 |  | 1900 | 4.5 | 3.2 | |
| 11-13 | 2350 | 4.5 | 3.2 |  | 2050 | 4.9 | 3.4 | |
| ≥14 | 2850 | 5.0 | 3.5 |  | 2300 | 5.0 | 3.8 | |

RNI, recommended nutrient intake.
