# Supplementary material for: Consistency between 3 days' dietary records and 24-h urine in estimating salt intake in children and adolescents
Source: Front Public Health. 2022 Dec 23;10:1071473. doi: 10.3389/fpubh.2022.1071473 (PMC9822649; doi:10.3389/fpubh.2022.1071473)
Supplement: Supplementary file 2 [file Table_2.DOCX]

Supplemental Table 2. Records of three-days’ salt and salty seasonings used in each food item in the three schools’ cafeterias ^a^.

^a^ Packaged food, including bread and bao, were not listed in the table.

Stable 2a. School 1 (FX).

|  | Net weight, kg | Seasonings, g ^a^ | | | | Sodium, g | Sodium concentration, mg/100g |
| --- | --- | --- | --- | --- | --- | --- | --- |
|  |  | Salt | MSG | Soy sauce | Hotpot broth base |  |  |
| Day 1 |  |  |  |  |  |  |  |
| Lunch |  |  |  |  |  |  |  |
| soup | 102 | 1031 | 10 |  |  | 406.7 | 398.7 |
| meat+veg1 | 64.6 | 1031 | 166.5 |  |  | 428.0 | 662.6 |
| meat+veg2 | 82.5 | 790.5 | 171.1 | 430.5 |  | 369.4 | 447.7 |
| veg | 50.4 | 416 | 107.5 |  |  | 178.2 | 353.5 |
| Dinner |  |  |  |  |  |  |  |
| soup | 47.6 | 324 | 80 |  |  | 138.3 | 290.5 |
| meat+veg1 | 30.45 | 200 | 19.5 |  | 778 | 112.4 | 369.2 |
| meat+veg2 | 32.75 | 340.9 | 97 |  |  | 147.2 | 449.6 |
| veg | 37.75 | 436.85 | 43.5 | 137 |  | 188.9 | 500.4 |
| Day 2 |  |  |  |  |  |  |  |
| Breakfast ^b^ | 53.15 | 274.1 | 81.2 | 150 |  | 131.1 | 246.7 |
| Lunch |  |  |  |  |  |  |  |
| soup | 101.96 | 1031 | 117 |  |  | 421.3 | 413.2 |
| meat+veg1 | 56.4 | 958.3 | 134 | 302 |  | 419.8 | 744.3 |
| meat+veg2 | 78.6 | 1031 | 97 | 292 |  | 442.5 | 563 |
| veg | 72.1 | 613.9 | 136 | 256 |  | 280.9 | 389.5 |
| Dinner |  |  |  |  |  |  |  |
| soup | 50.65 | 515.5 | 58.1 |  |  | 210.6 | 415.8 |
| meat+veg1 | 58.45 | 515.5 | 55.1 | 284 |  | 233.5 | 399.4 |
| meat+veg2 | 23.45 | 443.1 | 41.5 |  |  | 179.9 | 767 |
| veg | 17.59 | 345.9 | 37.1 |  |  | 141.0 | 801.8 |
| Day 3 |  |  |  |  |  |  |  |
| Lunch |  |  |  |  |  |  |  |
| soup | 102.05 | 1031 | 119 |  |  | 421.5 | 413.1 |
| meat+veg1 | 72.9 | 1031 | 277 | 376.5 |  | 473.9 | 650.1 |
| meat+veg2 | 87.95 | 1031 | 160 | 413.5 |  | 461.0 | 524.2 |
| veg | 48.75 | 1031 | 263 |  |  | 441.1 | 904.9 |
| Dinner |  |  |  |  |  |  |  |
| soup | 54.75 | 515.5 | 95 |  |  | 215.6 | 393.8 |
| meat+veg1 | 44.2 | 515.5 | 161 |  |  | 224.6 | 508.1 |
| meat+veg2 | 35.55 | 515.5 | 209 |  |  | 231.1 | 650.1 |
| veg | 24.5 | 515.5 | 63 |  |  | 211.2 | 862.2 |

Note, ^a^ proportion of sodium in salt is 0.393; proportion of sodium in MSG is 0.136; proportion of sodium in soy sauce is 0.082; proportion of sodium in hotpot broth base is 0.04. ^b^ the say sauce for rice noodles. MSG, monosodium glutamate; veg, vegetable.

Stable 2b. School 2 (PX).

|  | Net weight, kg | Seasonings, g ^a^ | | | | | | | Sodium, g | Sodium concentration，mg/100g |
| --- | --- | --- | --- | --- | --- | --- | --- | --- | --- | --- |
|  |  | Salt | MSG | Light soy sauce | Dark soy sauce | Vine  -gar | Cooking wine | Hotpot broth base |  |  |
| Day 1 |  |  |  |  |  |  |  |  |  |  |
| Breakfast ^b^ | 82.3 | 803 | 72.8 | 658 | 128 |  |  |  | 378.3 | 459.6 |
| Lunch |  |  |  |  |  |  |  |  |  |  |
| soup | 156 | 160 | 15.8 |  |  |  |  |  | 64.8 | 41.6 |
| meat+veg1 | 63.3 | 706 |  | 658 | 500 |  | 1000 | 600 | 392.6 | 620.2 |
| veg | 122.4 | 867.5 |  | 1879.5 |  | 98.5 |  |  | 463.7 | 378.8 |
| Dinner |  |  |  |  |  |  |  |  |  |  |
| soup | 73.05 | 136.5 | 7.9 |  |  |  |  |  | 54.6 | 74.7 |
| meat+veg1 | 23.7 | 377.1 |  |  |  |  |  |  | 147.8 | 623.4 |
| meat+veg2 | 31 | 180.5 |  |  |  |  |  |  | 70.7 | 228.1 |
| veg | 14.3 | 119.2 |  |  |  |  |  |  | 46.7 | 326.5 |
| Day 2 |  |  |  |  |  |  |  |  |  |  |
| Breakfast ^b^ | 79.65 | 546.5 | 90.4 | 508 | 352.5 | 33.5 |  |  | 288.8 | 362.6 |
| Lunch |  |  |  |  |  |  |  |  |  |  |
| soup | 156 | 160 | 15.8 |  |  |  |  |  | 64.8 | 41.6 |
| meat+veg1 | 91.95 | 568 |  | 1386 | 500 | 20 | 1000 | 600 | 386.4 | 420.2 |
| veg | 78.36 | 913.1 |  |  |  |  |  |  | 357.8 | 456.6 |
| Dinner |  |  |  |  |  |  |  |  |  |  |
| soup | 73.05 | 250.6 |  |  |  |  |  |  | 98.2 | 134.4 |
| meat+veg1 | 46.3 | 165 |  |  |  |  |  |  | 64.7 | 139.6 |
| veg | 35.2 | 143.6 |  |  |  |  |  |  | 56.3 | 159.8 |
| Day 3 |  |  |  |  |  |  |  |  |  |  |
| Breakfast ^b^ | 79.15 | 363.6 | 40 | 937 | 192 | 55.5 |  |  | 225.4 | 284.7 |
| Lunch |  |  |  |  |  |  |  |  |  |  |
| soup | 156 | 160 | 15.8 |  |  |  |  |  | 64.8 | 41.6 |
| meat+veg1 | 72.75 | 500 |  | 1386 | 500 | 20 | 1000 | 600 | 359.8 | 494.5 |
| meat+veg2 | 30.8 | 298 |  |  |  |  |  |  | 116.8 | 378.8 |
| veg | 43 | 358.4 |  |  |  |  |  |  | 140.4 | 326.5 |
| Dinner |  |  |  |  |  |  |  |  |  |  |
| soup | 60.05 | 22.7 |  |  |  |  |  |  | 8.9 | 14.8 |
| meat+veg1 | 62.8 | 260 |  |  |  |  |  |  | 101.8 | 162.1 |
| veg | 51.7 | 365 |  |  |  |  |  |  | 143 | 276.6 |

Note, ^a^ proportion of sodium in salt is 0.392; proportion of sodium in MSG is 0.136; proportion of sodium in light soy sauce is 0.066; proportion of sodium in dark soy sauce is 0.082; proportion of sodium in vinegar is 0.0023; proportion of sodium in cooking wine is 0.0009; proportion of sodium in hotpot broth base is 0.0513. ^b^ rice noodles with soy sauce. MSG, monosodium glutamate; veg, vegetable.

Stable 2c. School 3 (PZ).

|  | Net weight, kg | Seasonings, g ^a^ | | | Sodium, g | Sodium concentration, mg/100g |
| --- | --- | --- | --- | --- | --- | --- |
|  |  | Salt | MSG | Soy sauce |  |  |
| Day 1 |  |  |  |  |  |  |
| Breakfast ^b^ | 28.12 | 301 | 180 | 367 | 169.8 | 603.9 |
| Lunch |  |  |  |  |  |  |
| soup | 47.5 | 288.1 | 122.5 |  | 129.9 | 273.6 |
| meat+veg1 | 41.85 | 252 | 139.3 | 461 | 150.9 | 360.6 |
| meat+veg2 | 85 | 649 | 171.7 |  | 278.5 | 327.7 |
| veg | 68.1 | 467 | 94.3 |  | 196.4 | 288.5 |
| Dinner |  |  |  |  |  |  |
| soup | 44.7 | 246.6 | 126.7 |  | 114.2 | 255.5 |
| meat+veg1 | 32.25 | 252 |  | 461 | 131.9 | 409.1 |
| meat+veg2 | 58.45 | 265.5 | 97.6 |  | 117.7 | 201.3 |
| veg | 70.65 | 531.5 | 172 |  | 232.4 | 328.9 |
| Day 2 |  |  |  |  |  |  |
| Lunch |  |  |  |  |  |  |
| soup | 46.5 | 307.9 | 111.5 |  | 136.2 | 293 |
| meat+veg1 | 35.1 | 252 | 110.7 | 461 | 147 | 418.8 |
| meat+veg2 | 65.6 | 389.5 |  |  | 153.1 | 233.4 |
| veg | 74.85 | 418.2 | 133 |  | 182.5 | 243.8 |
| Dinner |  |  |  |  |  |  |
| soup | 47.4 | 187 | 207 |  | 101.7 | 214.5 |
| meat+veg1 | 42.4 |  | 178 |  | 24.2 | 757.1 |
| meat+veg2 | 72.7 | 305 | 159 |  | 141.6 | 194.7 |
| veg | 65 | 139.3969 |  |  | 54.8 | 84.3 |
| Day 3 |  |  |  |  |  |  |
| Lunch |  |  |  |  |  |  |
| soup | 42.9 | 74.8 | 59.8 |  | 37.5 | 87.5 |
| meat+veg1 | 34.4 | 252 | 64.2 | 461 | 140.7 | 409 |
| meat+veg2 | 66.6 | 406 | 176.1 |  | 183.6 | 275.7 |
| veg | 64.1 | 575 | 71.6 | 33.5 | 238.2 | 371.6 |
| Dinner |  |  |  |  |  |  |
| soup | 43.6 | 246.5 | 76 |  | 107.3 | 246 |
| meat+veg1 | 58 | 257.5 | 59 | 129.5 | 118.5 | 204.3 |
| meat+veg2 | 84.3 | 500 | 175 |  | 220.4 | 261.4 |
| veg | 65.5 |  | 139.6 |  | 19 | 429 |

Note, ^a^ proportion of sodium in salt is 0.393; proportion of sodium in MSG is 0.136; proportion of sodium in soy sauce is 0.071. ^b^ the say sauce for rice noodles. MSG, monosodium glutamate; veg, vegetable.
