# Supplementary material for: Consistency between 3 days' dietary records and 24-h urine in estimating salt intake in children and adolescents
Source: Front Public Health. 2022 Dec 23;10:1071473. doi: 10.3389/fpubh.2022.1071473 (PMC9822649; doi:10.3389/fpubh.2022.1071473)
Supplement: Supplementary file 3 [file Table_3.DOCX]

Supplemental Table 3. Net intake and sodium intake of each food item in the three days in the three schools.

Stable 3a. School 1 (FX).

|  | Cna, mg/100g | n | Median intake, g | Sodium intake, mg |
| --- | --- | --- | --- | --- |
| Day 1 |  |  |  |  |
| Breakfast |  |  |  |  |
| congee | 0 | 30 | 200 [200, 400] | 0 |
| boiled egg | 0 | 25 | 50 | 0 |
| bread | 181 | 33 | 100 [85, 100] | 181 [145, 181] |
| Total |  | 37 |  | 181 [109, 181] |
| Lunch |  |  |  |  |
| soup | 398.7 | 54 | 115 [100, 150] | 459 [399, 598] |
| meat+veg1 | 662.6 | 54 | 50 [40, 90] | 331 [265, 596] |
| meat+veg2 | 447.7 | 54 | 50 [40, 90] | 224 [179, 403] |
| veg | 353.5 | 54 | 50 [40, 78] | 177 [141, 234] |
| rice |  | 54 | 193 [174, 206] | 0 |
| Total |  | 54 |  | 1243 [990, 1599] |
| Dinner |  |  |  |  |
| soup | 290.5 | 54 | 160 [130, 182] | 465 [352, 529] |
| meat+veg1 | 369.2 | 54 | 22 [11, 69] | 97 [48, 309] |
| meat+veg2 | 449.6 | 54 | 69 [27, 106] | 255 [99, 392] |
| veg | 500.4 | 54 | 18 [8, 57] | 73 [40, 285] |
| rice |  | 54 | 238 [138, 263] | 0 |
| Total |  | 54 |  | 889 [677, 1297] |
| Day 2 |  |  |  |  |
| Breakfast |  |  |  |  |
| rice noodle | 246.7 ^a^ | 40 | 477 [281, 653] ^b^ | 353 [205, 464] |
| steamed bread | 0 | 18 ^c^ |  | 0 |
| Total |  | 40 |  | 353 [205, 464] |
| Lunch |  |  |  |  |
| soup | 413.2 | 54 | 140 [90, 140] | 578 [372, 578] |
| meat+veg1 | 744.3 | 54 | 20 [7, 52] | 145 [50, 383] |
| meat+veg2 | 563 | 54 | 55 [37, 114] | 310 [208, 642] |
| veg | 389.5 | 54 | 26 [10, 45] | 95 [39, 155] |
| rice |  | 54 | 181 [132, 203] | 0 |
| Total |  | 54 |  | 1138 [846, 1523] |
| Dinner |  |  |  |  |
| soup | 415.8 | 54 | 144 [120, 159] | 597 [497, 661] |
| meat+veg1 | 399.4 | 54 | 93 [60, 114] | 369 [230, 455] |
| meat+veg2 | 767 | 54 | 45 [28, 79] | 345 [176, 606] |
| veg | 801.8 | 54 | 38 [16, 75] | 301 [126, 601] |
| rice |  | 54 | 208 [185, 248] | 0 |
| Total |  | 54 |  | 1593 [1157, 2313] |
| Day 3 |  |  |  |  |
| Breakfast |  |  |  |  |
| congee | 0 | 31 | 200 [200, 200] | 0 |
| boiled egg | 0 | 25 | 50 | 0 |
| bread | 181 | 33 | 100 [100, 100] | 181 [181, 181 ] |
| Total |  | 35 |  | 181 [181, 181] |
| Lunch |  |  |  |  |
| soup | 413.1 | 51 | 150 [150, 150] | 620 [620, 620] |
| meat+veg1 | 650.1 | 51 | 87 [29, 97] | 566 [189, 631] |
| meat+veg2 | 524.2 | 51 | 50 [27, 112] | 262 [142, 587] |
| veg | 904.9 | 51 | 49 [28, 92] | 443 [253, 833] |
| rice |  | 51 | 222 [192, 299] | 0 |
| Total |  | 51 |  | 1916 [1458, 2449] |
| Dinner |  |  |  |  |
| soup | 393.8 | 53 | 200 [184, 205] | 788 [725, 807] |
| meat+veg1 | 508.1 | 53 | 119 [62, 145] | 605 [312, 737] |
| meat+veg2 | 650.1 | 53 | 54 [30, 115] | 351 [192, 748] |
| veg | 862.2 | 53 | 31 [12, 96] | 267 [86, 823] |
| rice | 0 | 53 | 204 [175, 228] | 0 |
| Total |  | 53 |  | 1758 [1581, 3054] |

Note, ^a^ the sodium concentration was only for the say sauce in rice noodles; ^b^ the net intake consisted of rice noodles and soy sauce; ^c^ the net weight of steamed bread was 50 g and 16 participants had one streamed bread and 2 participants had two steamed bread. Cna, sodium concentration in mg/100g; veg, vegetable.

Stable 3b. School 2 (PX).

|  | Cna, mg/100g | n | Net intake, g | Sodium intake, mg |
| --- | --- | --- | --- | --- |
| Day 1 |  |  |  |  |
| Breakfast |  |  |  |  |
| rice noodle | 459.6 | 39 | 300 [238, 356] | 1379 [1094, 1636] |
| congee | 0 | 11 | 278 [259, 297] | 0 |
| steamed bread | 0 | 11 | 83 [80, 119] | 0 |
| Total |  | 51 |  | 1163 [285, 1620] |
| Lunch |  |  |  |  |
| soup | 41.6 | 56 | 169 [146, 190] | 70 [61, 79] |
| meat+veg1 | 620.2 | 56 | 105 [89, 115] | 651 [552, 713] |
| veg | 378.8 | 56 | 111 [101, 120] | 419 [383, 453] |
| rice | 0 | 56 | 257 [237, 349] | 0 |
| Total |  | 56 |  | 1146 [1052, 1199] |
| Dinner |  |  |  |  |
| soup | 74.7 | 55 | 180 [155, 205] | 134 [116, 153] |
| meat+veg1 | 623.4 | 55 | 91 [46, 115] | 564 [284, 717] |
| meat+veg2 | 228.1 | 55 | 65 [43, 86] | 147 [98, 196] |
| veg | 326.5 | 55 | 71 [44, 87] | 232 [144, 284] |
| rice | 0 | 55 | 233 [215, 250] | 0 |
| Total |  | 55 |  | 1064 [776, 1318] |
| Day 2 |  |  |  |  |
| Breakfast |  |  |  |  |
| rice noodle | 362.6 | 36 | 307 [254, 349] | 1111 [919, 1264] |
| congee | 0 | 9 | 200 [125, 259] | 0 |
| steamed bread | 0 | 11 | 115 [61, 122] | 0 |
| brown sugar steamed bread | 0 | 13 | 130 [91, 163] | 0 |
| Total |  | 51 |  | 943 [0, 1218] |
| Lunch |  |  |  |  |
| soup | 41.6 | 57 | 133 [124, 149] | 55 [52, 62] |
| meat+veg1 | 420.2 | 57 | 126 [114, 144] | 529 [477, 605] |
| veg | 456.6 | 57 | 92 [80, 106] | 420 [363, 484] |
| rice | 0 | 57 | 228 [202, 264] | 0 |
| Total |  | 57 |  | 1022 [895, 1135] |
| Dinner |  |  |  |  |
| soup | 134.4 | 57 | 163 [147, 174] | 219 [197, 233] |
| meat+veg1 | 139.6 | 57 | 114 [90, 134] | 159 [125, 187] |
| veg | 159.8 | 57 | 98 [86, 123] | 157 [137, 197] |
| rice | 0 | 57 | 267 [238, 296] | 0 |
| Total |  | 57 |  | 533 [452, 585] |
| Day 3 |  |  |  |  |
| Breakfast |  |  |  |  |
| rice noodle | 284.7 | 40 | 290 [198, 379] | 824 [562, 1078] |
| congee | 0 | 7 | 178 [123, 258] | 0 |
| steamed bread | 0 | 12 | 129 [94, 144] | 0 |
| brown sugar steamed bread | 0 | 3 | 88/126/127 | 0 |
| Total |  | 50 |  | 726 [321, 1006] |
| Lunch |  |  |  |  |
| soup | 41.6 | 57 | 156 [124, 178] | 65 [51, 74] |
| meat+veg1 | 494.5 | 57 | 104 [49, 124] | 514 [242, 611] |
| meat+veg2 | 378.8 | 57 | 106 [72, 121] | 402 [271, 456] |
| veg | 326.5 | 57 | 99 [75, 118] | 323 [245, 385] |
| rice | 0 | 57 | 213 [191, 242] | 0 |
| Total |  | 57 |  | 1338 [873, 1472] |
| Dinner |  |  |  |  |
| soup | 14.8 | 57 | 140 [115, 157] | 21 [17, 23] |
| meat+veg1 | 162.1 | 57 | 136 [116, 145] | 221 [187, 235] |
| veg | 276.6 | 57 | 122 [85, 133] | 337 [235, 368] |
| rice | 0 | 57 | 196 [173, 267] | 0 |
| Total |  | 57 |  | 576 [462, 617] |

Cna, sodium concentration in mg/100g; veg, vegetable.

Stable 3c. School 3 (PZ).

|  | Cna, mg/100g | n | Median intake, g | Sodium intake, mg |
| --- | --- | --- | --- | --- |
| Day 1 |  |  |  |  |
| Breakfast |  |  |  |  |
| rice noodle | 603.9 ^a^ | 5 | 353 [164, 491] ^b^ | 775 [669, 1094] |
| congee | 0 | 16 | 150 [150, 200] | 0 |
| steamed meat bun | 212 | 22 | 86 [86, 172] | 182 [182, 430] |
| Total |  | 22 | - | 182 [182, 430] |
| Lunch |  |  |  |  |
| soup | 273.6 | 32 | 78 [43, 150] | 212 [117, 410] |
| meat+veg1 | 360.6 | 32 | 63 [39, 109] | 225 [142, 391] |
| meat+veg2 | 327.7 | 32 | 119 [83, 136] | 388 [271, 445] |
| veg | 288.5 | 32 | 109 [86, 138] | 314 [247, 397] |
| rice |  | 32 | 233 [199, 259] | 0 |
| Total |  | 32 | - | 1176 [956, 1354] |
| Dinner |  |  |  |  |
| soup | 255.5 | 32 | 122 [76, 156] | 310 [194, 397] |
| meat+veg1 | 409.1 | 32 | 68 [47, 102] | 278 [192, 416] |
| meat+veg2 | 201.3 | 32 | 123 [93, 142] | 248 [187, 285] |
| veg | 328.9 | 32 | 146 [91, 160] | 480 [298, 525] |
| rice |  | 32 | 241 [215, 316] | 0 |
| Total |  | 32 | - | 1291 [984, 1585] |
| Day 2 |  |  |  |  |
| Breakfast |  |  |  |  |
| rice noodle | 603.9 ^a^ | 16 | 253 [153, 639] ^b^ | 746 [475, 959] |
| congee | 0 | 14 | 190 [160, 325] | 0 |
| steamed bread | 0 | 12 | 110 [100, 196] | 0 |
| Total |  | 28 | - | 419 [0, 841] |
| Lunch |  |  |  |  |
| soup | 293 | 32 | 110 [55, 122] | 321 [160, 357] |
| meat+veg1 | 418.8 | 32 | 83 [55, 119] | 348 [228, 499] |
| meat+veg2 | 233.4 | 32 | 103 [73, 134] | 240 [169, 313] |
| veg | 243.8 | 32 | 142 [115, 155] | 345 [281, 378] |
| rice |  | 32 | 259 [233, 333] | 0 |
| Total |  | 32 | - | 1214 [978, 1509] |
| Dinner |  |  |  |  |
| soup | 214.5 | 32 | 131 [124, 141] | 281 [267, 301] |
| meat+veg1 | 757.1 | 32 | 125 [107, 130] | 946 [810, 984] |
| meat+veg2 | 194.7 | 32 | 136 [94, 148] | 265 [184, 288] |
| veg | 84.3 | 32 | 123 [77, 133] | 103 [65, 112] |
| rice |  | 32 | 242 [222, 333] | 0 |
| Total |  | 32 | - | 1586 [1323, 1694] |
| Day 3 |  |  |  |  |
| Breakfast |  |  |  |  |
| rice noodle | 603.9 ^a^ | 14 | 437 [258, 560] ^b^ | 696 [615, 1108] |
| congee | 0 | 10 | 200 [200, 250] | 0 |
| steamed bread | 0 | 9 | 105 [98, 211] | 0 |
| Total |  | 26 | - | 516 [0, 714] |
| Lunch |  |  |  |  |
| soup | 87.5 | 32 | 130 [91, 162] | 113 [80, 142] |
| meat+veg1 | 409 | 32 | 106 [63, 125] | 431 [258, 511] |
| meat+veg2 | 275.7 | 32 | 140 [83, 152] | 385 [229, 418] |
| veg | 371.6 | 32 | 150 [118, 177] | 556 [438, 657] |
| rice |  | 32 | 248 [215, 375] | 0 |
| Total |  | 32 | - | 1423 [1016, 1714] |
| Dinner |  |  |  |  |
| soup | 246 | 32 | 128 [117, 144] | 315 [288, 354] |
| meat+veg1 | 204.3 | 32 | 183 [156, 199] | 374 [318, 406] |
| meat+veg2 | 261.4 | 32 | 151 [137, 168] | 393 [358, 439] |
| veg | 429 | 32 | 116 [108, 131] | 498 [464, 561] |
| rice | 0 | 32 | 254 [221, 475] | 0 |
| Total |  | 32 | - | 1612 [1495, 1706] |

Note, ^a^ the sodium concentration was only for the say sauce in rice noodles; ^b^ the net intake consisted of rice noodles and soy sauce. Cna, sodium concentration in mg/100g; veg, vegetable.
